# Antimicrobial Susceptibility of Bacterial Isolates from Donkey Uterine Infections, 2018–2021

**DOI:** 10.3390/vetsci9020067

**Published:** 2022-02-05

**Authors:** Yufei Zhao, Yiping Zhu, Bo Liu, Junpeng Mi, Nan Li, Weisen Zhao, Rongzheng Wu, Gilbert Reed Holyoak, Jing Li, Dejun Liu, Shenming Zeng, Yang Wang

**Affiliations:** 1 College of Veterinary Medicine, China Agriculture University, Beijing 100193, China; sy20203050874@cau.edu.cn (Y.Z.); yipingz@cau.edu.cn (Y.Z.); bliu2017@cau.edu.cn (B.L.); sy20203050883@cau.edu.cn (N.L.); witch@cau.edu.cn (R.W.); wangyang@cau.edu.cn (Y.W.); 2 School of Veterinary Science, University of Sydney, Sydney, NSW 2000, Australia; jumi0731@uni.sydney.edu.au; 3 College of Animal Science and Technology, China Agriculture University, Beijing 100193, China; sy20203050871@cau.edu.cn (W.Z.); zengsm@cau.edu.cn (S.Z.); 4 College of Veterinary Medicine, Oklahoma State University, Stillwater, OK 74078, USA; reed.holyoak@okstate.edu

**Keywords:** endometritis, subfertility, donkeys, prevalence, bacteriology, antimicrobial susceptibility, MDR

## Abstract

Background: Endometritis is a common reproductive disease in equine animals. No investigation about the bacterial characteristics and antimicrobial susceptibility pattern of donkeys with endometritis has thus far been reported. Objectives: To determine the common uterine bacterial isolates from donkeys with endometritis and to evaluate their susceptibility to antimicrobials used for the treatment thereof. Study design: Retrospective case-series. Methods: Medical records at an equine clinical diagnostic center were retrospectively reviewed to identify submissions from donkeys with bacterial endometritis between 2018 and 2021. Data were extracted and analyzed descriptively in terms of the frequency of bacterial species, susceptibility to antimicrobials and multidrug resistance. Results: A total of 73 isolates were identified from 30 donkeys, of which 92% of the isolates were Gram-negative bacteria. Mixed cultures were found in 90% of the donkeys. The most common isolates were *Escherichia* *coli* (31.5%) and *Acinetobacter* spp. (21.9%). Susceptibility testing revealed that amikacin (98%), cefoxitin (95%), trimethoprim-sulfamethoxazole (78%) and gentamicin (74%) were the most efficient agents for donkeys. Multidrug resistance (MDR) was found in 20% of all bacterial isolates, of which all *Pseudomonas aeruginosa* isolates showed a multidrug resistance profile. Main limitations: The sample size was relatively small, which means a bias of selection may exist. The antimicrobial resistance and MDR of agents without break points were not calculated, which means the relative results may be underestimated in our study. Conclusions: Severe infections were detected in donkeys with endometritis. Antimicrobial resistance and MDR bacteria are not rare in our study. This study demonstrated that bacteria identification and antimicrobial susceptibility testing are highly recommended before the treatment of uterine infections in donkeys. Further studies, including the epidemiological investigation of bacterial endometritis of donkeys, should be conducted to provide a better understanding of this critical problem.

## 1. Introduction

The Nubian donkey (*E. asinus africanus*) and the Somali donkey (*E. asinus somaliensis*) were two original ancestors of today’s donkey species (*Equus Asinus*). These species accompanied the development of human civilization from Africa to Europe and Asia, over 10,000 years ago [1]. Donkey breeds and breeding populations have declined worldwide over the past century, due to a loss of their roles in human society. However, there has been a renewed interest and demand for donkeys (*E. Asinus*) in recent years, for their novel and evolving role in meat, milk, and skin production [2]. Increasing the reproductive efficiency of donkeys is essential for achieving sustainable populations and economic value.

Endometritis is the third most common equine disease [3], and has long been recognized as one of the major concerns of the equine breeding industry [3,4]. Although endometritis can be associated with a number of causes, bacterial infection, especially aerobic bacteria, is regarded as the main cause of endometritis [5], occurring in 25% to 60% of mares with infertility [6,7]. In mares, retrospective studies reported in several regions over the years have shown that *Streptococcus equi subsp zooepidemicus (SEZ)*, *Escherichia coli (E. coli)*, *Klebsiella pneumoniae (K. pneumoniae)*, and *Pseudomonas aeruginosa (P. aeruginosa)* are the most common pathogenic microorganisms of bacterial endometritis [8,9]. This disease has caused significant financial losses, mainly due to failed conception and early embryonic death [7,10].

The presence of an endometrial microbiome in the equine uterus has been reported. The endometrial microbiome refers to the genome of the microorganisms that were discovered from endometrial samples. Over 200 bacterial species have been discovered from equine endometrial samples in recent years [11,12,13]. The potential connection between intrauterine and extrauterine microbiomes make it possible for pathogenic microorganisms to enter the uterus by various means, including mating, artificial insemination or urogenital veterinary examinations, and cervical defects or other failures of physical barriers to infection [7,14]. Transient endometritis can be seen as a normal physiological reaction of mares to eliminate pathogenic bacteria from the uterine lumen [15,16]. Physiologically, mares can autonomously clear the intrauterine bacteria and inflammatory responses within 48 h, while the “susceptible” mares may fail to do so, leading to dysbiotic bacterial endometritis [17].

The consensus statement of antimicrobial drugs used in veterinary medicine has recommended that any use of antimicrobials should be based on the results of laboratory bacteriology and antimicrobial susceptibility testing [18]. Meanwhile, empirical antibiotic treatments based on early studies are often administrated without laboratory diagnoses in the clinic [19], as laboratory diagnoses often take several days, and equine practitioners need to treat infections adequately while the animal is still in estrus [20]. In such cases, treatments often have a lower than desired efficacy. Several studies have been reported on horses confirming that pathogenic species and their antimicrobial susceptibility patterns vary greatly over time and across geographic locations [19,21,22,23,24].

Donkeys share many similarities with horses in reproductive features. For example, the length of uterine body to uterine horn are similar in donkeys and mares, and the shape and structure of the ovaries also resemble each other. Yet, donkeys have longer and narrower cervixes, and uterine edema are less commonly seen in donkeys than in mares [2]. From a reproductive physiology aspect, mares are a seasonal polyestrous animal, while donkeys are a non-seasonal species. In mares, two follicular waves can be seen before ovulation, whereas in donkeys, there appears to be only one follicular wave [2]. Differences here make it even more unreliable to treat endometritis in donkeys with data referenced exclusively from horses. As far as we can assess, information on bacteria prevalence and the antimicrobial susceptibility of the causative organisms of endometritis in donkeys is sparse, and there is insufficient information to provide a valid clinical reference for empirical treatment.

The aim of this study was to identify the bacterial characteristics in samples submitted from donkeys with endometritis, and the antimicrobial susceptibility patterns of the isolated bacteria. A potential goal of reporting these data is to establish a preliminary basis for the empirical treatment of endometritis in donkeys.

## 2. Materials and Methods

### 2.1. Data Collection

Medical records of submitted uterine samples obtained from donkeys that had positive bacteriologic culture, cytological evaluation, and antimicrobial susceptibility results to the Equine Clinical Diagnostic Center (ECDC), China Agricultural University, between 1 June 2018 and 15 June 2021, were reviewed. Thirty donkeys with a clinical diagnosis of bacterial endometritis were included and sampled. Breeds represented included “Yangyuan donkeys” (*n* = 27) and “Dezhou donkeys” (*n* = 3). The average age was 4.1 years (range, 2 to 7 years old). Among the donkeys included, 14 donkeys (14/30, 46.7%) showed purulent vulvar/vulvovaginal discharge; intrauterine fluid was detected by ultrasonography from 16 donkeys’ uterine (16/30, 53.3%). All included donkeys were nulliparous, and they were all reported to have failed artificial insemination with fresh semen within a year.

According to the protocols of ECDC, all samples were collected by double-guarded uterine swabs. To avoid contamination as much as possible, the donkey’s perineum and vulva were washed with soap and water until clean. When sampling, the operator was asked to wear sterile rectal examination gloves (Jiangs, Nanning, China) and use disposable double-guarded swabs (IMV, Legler, France) to collect endometrial samples [25,26]. Each sample was immediately placed into Amies transport medium (Hopebio, Qingdao, China) and sent back to ECDC laboratory at room temperature within 24 h [19,22]. Other signalments such as age, breed, estrus period and pregnancy history of the included donkeys were also extracted. A written consent for the use of the data was obtained from each owner.

### 2.2. Bacterial Culturing and Identification

In the ECDC laboratory, bacterial culture and isolation procedures were carried out in accordance with Clinical and Laboratory Standards Institute (CLSI) documents M100-ED30 and VET04 [27,28]. Swabs sampled were streaked directly on Columbia blood agar (CBA) and MacConkey agar (Land Brigde, Beijing, China), intended for the culture of aerobic bacteria. The agar plates were incubated at 37 °C and examined at 24 h and 48 h to obtain individual colonies [22]. The microorganisms obtained were initially identified by the morphology of colonies, time of growth, hemolysis on the plates, and Gram stain microbiology. Bacteria growth was reported as slight (10–20 colony forming unit (CFU)/plate), moderate (20–100 CFU/plate), or abundant (>100 CFU/plate). Further identifications were performed by both matrix-assisted laser desorption ionization–time-of-flight mass spectrometry (MALDI-TOF MS), and polymerase chain reaction (PCR) [29,30].

### 2.3. Antimicrobial Susceptibility Test

Based on CLSI guidelines [27,28], an antimicrobial susceptibility test (AST) was performed using broth microwell dilution. The antimicrobials tested were amoxicillin/clavulanic acid, cefazolin, cefoxitin, ceftiofur, cefepime, gentamicin, amikacin, kanamycin, tetracycline, tigecycline, enrofloxacin, trimethoprim-sulfamethoxazole (TMPS), meropenem and rifampicin. Erythromycin and vancomycin were used only for Gram-positive bacteria. Based on the European Committee on Antimicrobial Sensitivity Testing’s (EUCAST) expert rules in antimicrobial susceptibility testing [31], if a certain bacterial isolate was intrinsically resistant to an antimicrobial, then this agent would not be evaluated for this bacteria species. After the bacterial isolates were suspended to approximately 5 × 10^5^ CFU/mL, they were added in cation-adjusted Mueller–Hinton broth (Land Brigde, Beijing, China), together with antimicrobials in 96-well panels, and incubated at 37 °C for 16–18 h. American Type Culture Collection (ATCC) bacterial strains were used for quality control.

The minimal inhibitory concentration (MIC) was recorded for each row on the panel. According to CLSI’s break points of the selected antimicrobials for each group of bacteria, the results of MIC were classified as sensitivity (S), intermediate (I) and resistant (R). When calculating susceptible rate, intermediate and resistant categories were grouped together. If breakpoints for equines were not available, human breakpoints were referred. Data were presented in MIC_50_ and MIC_90_ when there was no break point to refer to, according to CLSI [27]. Multi-drug resistance (MDR) was also recorded if one bacterial isolate was considered resistant to three or more antimicrobials [32].

### 2.4. Statistical Analysis

Basic data including clinical signalment (age, breed, clinical sign, history of pregnant, treatment), bacterial identification results, susceptibility results and endometrial cytological evaluation were documented in EXCEL (Microsoft, Redmond, WA, USA) to analyze the bacterial isolation rate, antimicrobial resistance rate and MDR rate. Descriptive analysis was performed by using the percentages and counts. The difference of relative frequencies between groups was compared using the Chi-square test and T test in R statistical computing software (Rstudio, Boston, MA, USA). For all comparisons, a value of *p* < 0.05 was considered significant.

## 3. Results

### 3.1. Bacteriologic Description

Of the 30 donkeys, three (3/30, 10%) yielded a single organism, while 27 (27/30, 90%) had two or more bacterial species cultured from submitted samples. In six donkeys (6/30, 20%), both Gram-positive and Gram-negative bacteria were identified. Twenty-four donkeys (24/30, 80%) had only Gram-negative isolates, while no donkeys (0%) yielded a Gram-positive isolate only. The complete bacterial isolation results are presented in Table 1.

A total of 73 bacterial isolates were obtained, of which six (6/73, 8%) were Gram-positive and 67 (67/73, 92%) were Gram-negative bacteria. The proportion of Gram-negative bacterial isolates was significantly higher than that of Gram-positive bacteria (*p* < 0.01). The most common isolated Gram-negative organism was *E.* *coli* (23/73, 31.5%), followed by *Acinetobacter* spp. (16/73, 21.9%) and *P.* *aeruginosa* (8/73, 11%). As for Gram-positive bacteria, *Streptococcus* spp. were the most common family, including three *SEZ* isolates (3/73, 4.1%).

*E. coli* was isolated from ten donkeys (10/14, 71.4%) with purulent vulvar discharge and from 13 donkeys (13/16, 81.3%) with intrauterine fluid detected by ultrasonography. *Acinetobacter* spp. was cultured from eight donkeys (8/14, 57.1%) with vulvar discharge and four donkeys (4/16, 25%) with intrauterine fluid. *P.* *aeruginosa* was only cultured from seven donkeys (7/14, 50%) with vulvar discharge. *SEZ* was isolated from two (2/14, 14.3%) donkeys with purulent vulvar discharge and one donkey (1/16, 6.3%) with fluid in intrauterine. *K.* *pneumonia* was cultured from two donkeys (2/16, 12.5%) with intrauterine fluid, while *Klebsiella oxytoca* was isolated from one donkey with discharge (1/14, 7.1%) and one donkey with fluid (1/16, 6.3%), respectively. The proportion of representative pathogenic bacterial isolates showed a significant difference between donkeys with vulvar discharge and those with intrauterine fluid (*p* < 0.05).

### 3.2. Antimicrobial Susceptibility Testing

Five species of isolated bacteria considered as potential pathogens of equine endometritis were included for antimicrobial susceptibility testing. Fifty strains of potentially pathogenic bacteria (50/73, 68.5%) isolated from 26 donkeys had antimicrobial susceptibility testing performed, yielding 19 antimicrobial susceptibility patterns. Complete results of AST are presented in Table 2 and Table 3. There was no break point of some antimicrobials, in those situations, MIC_50_ and MIC_90_ values were given. For example, cefoxitin MICs values showed <0.25 μg/mL to *SEZ* and >128 μg/mL to *P.* *aeruginosa* isolates. Kanamycin MICs values showed 32 μg/mL to *SEZ*, while they showed 128 μg/mL to *P.* *aeruginosa*. Complete results of these data are presented in Table 4.

Both cefepime and meropenem had the highest antimicrobial susceptibility frequencies, with all bacterial isolates (100%) being susceptible to them. Amikacin (98%), cefoxitin (95%), trimethoprim-sulfamethoxazole (78%) and gentamicin (74%) were also efficient agents. In addition to cefepime and meropenem, for *E. coli*, the proportion of resistant isolates was greatest for cefazolin (13%) and tetracycline (13%). All 23 *E. coli* isolates (100%) were classified as susceptible to cefoxitin, amikacin, and kanamycin. For the 13 *Acinetobacter* spp. isolates (including nine *Acinetobacter lwoffii (A. lwoffii)* isolates, three *Acinetobacter schindleri (A. schindleri)* isolates, and one *Acinetobacter baumannii (A. baumannii)* isolate), resistance was most common to cefazolin (84.6%). All the isolates (100%) were classified as susceptible to aminoglycosides. The seven *P.* *aeruginosa* isolates were classified as not susceptible to most of the selected antibacterial agents, except amikacin (100%) and TMPS (85.7%). For the three *SEZ* isolates, the proportion of resistant isolates was greatest to gentamicin (66.7%). They were classified as susceptible to all the other antimicrobials.

Antimicrobial susceptibility patterns revealed that 19 (19/26, 73.1%) of these donkeys had bacterial isolates resistant to the tested antimicrobials. Ten (10/12, 83.3%) had isolates resistant to cefazolin, and nine (9/12, 75%) had isolates resistant to gentamicin. Resistance to amoxicillin/clavulanic acid was found in seven donkeys (7/12, 58.3%). Five donkeys (5/12, 41.7%) had bacteria isolates resistant to kanamycin, while other isolates were resistant to tetracycline (3/12, 25%), ceftiofur (2/12, 16.7%), cefoxitin (1/12, 8.3%), tigecycline (1/12, 8.3%) and TMPS (2/12, 16.7%), respectively.

Of the donkeys with intra uterine fluid, seven (7/14, 50%) had bacterial isolates resistant to cefazolin. Some bacteria isolated from a few donkeys were also resistance to amoxicillin/clavulanic acid (2/14, 14.3%), gentamicin (1/14, 7.1%) and tetracycline (1/14, 7.1%). There was a significant difference (*p* < 0.05) found in the frequency of resistance for amoxicillin/clavulanic acid and gentamicin between donkeys with vulvar discharge and intrauterine fluid.

### 3.3. Multidrug Resistance

Ten (10/50, 20%) bacteria isolates were found to be multidrug-resistant based on the results of antimicrobial susceptibility testing. All the MDR bacteria found in this study were Gram-negative bacteria, including seven strains of *P.* *aeruginosa* (7/50, 14%), two strains of *E.* *coli* (2/50, 4%) and one strain of *A.* *lwoffii* (1/50, 2%).

Of the 26 donkeys that had antimicrobial susceptibility testing performed, nine (9/26, 34.6%) were infected with multidrug-resistant bacteria, all of them were those donkeys with purulent vulvar discharge. One donkey (1/26, 3.8%) with discharge was infected with more than one MDR bacteria. The frequency of MDR was more significant in donkeys with vulvar discharge than those with intrauterine fluid in the uterine (*p* = 0.011).

## 4. Discussion

To the best of our knowledge, this is the first reported antimicrobial susceptibility information from uterine infections of donkeys. The species and proportion of bacteria isolated in our study were different from those reported in mares [16,17,19,21,23]. Most of the included donkey samples (90%) yielded a mixed culture. Larger scale epidemiological investigations should be conducted in the future to increase the number of donkeys. Different geographical locations, the population of animals included, and the antimicrobials used before have been suggested to be the general causes [21,24].

*E. coli* and *Acinetobacter* spp. were the most common pathogenic bacterial species in this study. The frequency of *E. coli* being isolated was consistent with previous results (27.9–30%) in mares [21,22,23], but was a little higher than some others (17.3–19.3%) [17,24]. *E. coli,* along with *P. aeruginosa* and *K. pneumoniae*, have been regarded as biofilm-producing microorganisms in equine endometritis [33,34,35]. All these species showed high prevalence in our study compared with previous studies reported in mares [16,17], implying that biofilm may be a potentially serious problem in endometritis in donkeys. For *Acinetobacter* spp., the *A. lwoffii* and *A. schindleri* were the predominant species. Though no retrospective study reported these two species obtained from the mare or donkey uterus, there are reports of these species isolated from the feces of both horses and donkeys [36,37]. Additionally, these have been isolated from catheters used to treat endometritis of equines [38,39]. A possible explanation may be that the fecal contaminants were introduced into the uterus during the breeding process, causing opportunistic infections in these donkeys.

Gram-positive bacteria were not common isolates in our study. We only identified three *SEZ* isolates, which have always been reported as the most common Gram-positive pathogen causing endometritis in mares [6,21,22]. In research conducted in the middle-east of China, the infection rate of *SEZ* in donkeys with endometritis was 57%, which was much higher than the current result [40]. In contrast, in a donkey vaginal bacterial microbiota study conducted in Portugal, the isolate rate of *SEZ* was only 2% (3/140) [41]. *SEZ* often colonize deeply in the endometrium, which may make it more difficult for the swab to collect them [42]. However, given that current studies have reported an inconsistent prevalence of *SEZ* using swabs for sampling, it is difficult to quantitatively determine the relative importance of *SEZ* in causing endometritis in donkeys [16,17,23]. Meanwhile, *Staphylococcus* *aureus*, as another identifiable pathogen in mares [16,23], was negative from the samples submitted from these donkeys.

While there is growing concern worldwide about the public health impact of antimicrobial resistance (AMR) in livestock [43,44], few studies have investigated the efficacy of antimicrobials used for infections of the equine reproductive tract and uterus [45]. To provide up-to-date data on donkeys, nineteen antimicrobials were used in our study. Cefepime and meropenem were the most effective agents, which were 100% effective against both Gram-positive and Gram-negative bacteria in our study. They are all used as first-line antimicrobials in human hospitals [46,47]. However, cefepime has proven to have adverse effects on the gastrointestinal tract of horses [48]; thus, the further evaluation of the pharmacokinetics of cefepime in donkeys prior to clinical administration is necessary. Although meropenem is safe for use in equine animals, it is regarded as the last resort for the treatment of infections [49]. Therefore, the public health significance of the two most effective antimicrobials outweighs their practical clinical application in this study.

For Gram-negative bacteria, amikacin (98%) and cefoxitin were highly effective. The results agree with those reported previously [24]. Considering the absolute predominance of Gram-negative bacteria among pathogens in this study, we cautiously recommend these drugs for the clinical management of endometritis in donkeys. Gentamicin and TMPS are also commonly used for the treatment of equine bacterial infections [22]. The susceptibility of gentamicin (74%) in our study was not as pronounced as has been reported before (83–96%) [16,19]. Although the use of gentamicin is still recommended, an increasing resistance to gentamicin, due to empirical treatment without the identification of antimicrobial susceptibility, should be noted. Our results for TMPS (78%) were similar to those reported with mares in Sweden (2003) and Italy (2008) [17,19], but higher than those in other European and American countries over the past ten years [16,21,24]. The often-empirical use of gentamicin and TMPS in the treatment of endometritis may lead to increasing resistance of equine endometritis pathogens to them [22]. However, in donkeys, it seems that gentamicin and TMPS still have good antibacterial activity.

Rifampicin seemed to be an undesirable agent in our study, though there was no break point to refer to. Gram-negative bacteria were more resistant to RIF, and in this respect our results were consistent with those of others [17,23]. Reports of RIF resistance began in the time period when it was used in combination with macrolides [50]. The mutation in the β subunit of bacterial RNA polymerase (RNAP) may attribute to the resistance of RIF in bacteria [51]. The first-generation of cephalosporins have been reported to be less effective [23,24], which was supported by our results in the case of cefazolin. It has been recommended to use a combination of enrofloxacin and polymyxins against extensive drug-resistant (XDR) *P.* *aeruginosa* [52].

Analysis of multi-drug resistant bacteria from the reproductive tract, especially the uterus, has not previously been reported, yet a general trend of increasing MDR bacteria has been reported over time [29,53]. The classical definition of MDR was followed in our study [32]. The most common MDR isolates in our study were identified as *P.* *aeruginosa*, which account for 70% of all MDR bacteria. The results here raise a concern and suggest an urgent need to re-evaluate current practices and empirical treatment on donkey farms. Furthermore, although the proportion of MDR bacteria showed no difference between donkeys with vulvar discharge and those with intrauterine fluid, the composition of bacterial isolates and the effective agents of the in vitro AST between the donkeys with two clinical signs were different. This re-emphasizes the importance of identifying antimicrobial susceptibility before treatment.

In general, the level of medical care of donkeys has yet to be improved. Considering the donkey population and the farm scale in our surrounding area, it is very likely that the number of donkeys with bacterial endometritis is higher than the number presented to the ECDC laboratory. Therefore, one limitation of the current study was the relatively small size of sampled donkeys. In addition, since the antimicrobial resistance and MDR of agents without break points were not calculated, the relative results may also be underestimated in our study.

In conclusion, severe infections were detected in donkeys with endometritis. Antimicrobial resistance and MDR bacteria are not rare in uterine infections in donkeys. This study demonstrated that bacteria identification and antimicrobial susceptibility testing are highly recommended before any treatment of uterine infections in donkeys. Further studies, including the epidemiological investigation of bacterial endometritis in donkeys, should be conducted to provide a better understanding of this critical problem.

## Figures and Tables

**Table 1:** Species and frequency of bacteria isolated from uterine swabs of 30 donkeys at ECDC between 2018 and 2021.

Micro-Organisms	Number of Isolates	Frequency of Isolates (%)
*Acinetobacter baumannii*	3	4.1
*Acinetobacter lwoffii*	10	13.7
*Acinetobacter schindleri*	3	4.1
*Other Acinetobacter* spp.	1	1.7
*Aeromonas* spp.	1	1.7
*Arthrobacter gandavensis*	1	1.7
*Arthrobacter koreensis*	1	1.7
*Bacillus cereus*	2	2.7
*Burkholderia cepacia*	2	2.7
*Corynebacterium* spp.	1	1.7
*Enterobacter* spp.	3	4.1
*Escherichia coli*	23	31.5
*Klebsiella pneumoniae*	2	2.7
*Klebsiella oxytoca*	2	2.7
*Pantoea agglomerans*	1	1.7
*Proteus mirabilis*	2	2.7
*Pseudomonas aeruginosa*	8	10.9
*Other Pseudomonas* spp.	4	5.4
*Streptococcus equi subsp. zooepidemicus*	3	4.1
Total	73	100

**Table 2:** Antimicrobial susceptibility of Gram-negative bacteria isolated from the uterus of 30 donkeys at ECDC between 2018 and 2021.

Gram-Negative Bacteria	*E. coli* (23 Isolates)			*Acinetobacter* spp. (13 Isolates)			*P. aeruginosa* (7 Isolates)			*Klebsiella* spp. (4 Isolates)		
Antimicrobials ^a^	S (%)	I (%)	R (%)	S (%)	I (%)	R (%)	S (%)	I (%)	R (%)	S (%)	I (%)	R (%)
Amoxicillin/clavulanic-acid	91.3	-	8.7	92.3	7.7	-	-	-	100	50	25	25
Cefazolin	65.3	21.7	13	7.7	7.7	84.6	-	-	100	25	-	75
Cefoxitin	100	-	-	92.3	-	7.7	M ^b^	-	-	100	-	-
Ceftiofur	95.7	-	4.3	92.3	-	7.7	M ^b^	-	-	100	-	-
Cefepime	100	-	-	100	-	-	100	-	-	100	-	-
Gentamicin	87	4.3	8.7	100	-	-	-	-	100	100	-	-
Amikacin	100	-	-	100	-	-	100	-	-	100	-	-
Kanamycin	100	-	-	100	-	-	M ^b^	-	-	75	25	-
Tetracycline	87	-	13	92.3	-	7.7	NA ^c^	NA ^c^	NA ^c^	75	25	-
Tigecycline	95.7	4.3	-	92.3	-	7.7	NA ^c^	NA ^c^	NA ^c^	100	-	-
Enrofloxacin	M ^b^	-	-	M ^b^	-	-	M ^b^	-	-	M ^b^	-	-
Trimethoprim-Sulfamethoxazole	95.7	4.3	-	92.3	-	7.7	85.7	-	14.3	100	-	-
Meropenem	100	-	-	100	-	-	100	-	-	100	-	-
Rifampicin	M ^b^	-	-	M ^b^	-	-	M ^b^	-	-	M ^b^	-	-

^a^, S = sensitive, I = intermediate, R = resistant. ^b^, M = only MIC_50_ and MIC_90_ value were provided. Data were presented in this way when there was no break point to refer to, according to CLSI [27]. ^c^, NA = not applicable. *P.* *aeruginosa* was intrinsically resistant to tetracyclines according to EUCAST. Antimicrobial susceptibility to these agents were not evaluated for *P. aeruginosa* [31].

**Table 3:** Antimicrobial susceptibility of *SEZ* isolates collected from the uteruses of 30 donkeys at ECDC between 2018 and 2021.

Gram-Positive Bacteria	*SEZ* (3 Isolates)		
Antimicrobials ^a^	S (%)	I (%)	R (%)
Amoxicillin/clavulanic-acid	100	-	-
Cefoxitin	M ^b^	-	-
Ceftiofur	100	-	-
Cefepime	100	-	-
Gentamicin	-	33.3	66.7
Amikacin	66.7	33.3	-
Kanamycin	M ^b^	-	-
Tigecycline	100	-	-
Erythromycin	100	-	-
Enrofloxacin	Mb	-	-
Trimethoprim-Sulfamethoxazole	100	-	-
Meropenem	100	-	-
Vancomycin	100	-	-
Rifampicin	100	-	-

^a^, S = sensitive, I = intermediate, R = resistant. ^b^, M = only MIC_50_ and MIC_90_ value were provided. Data were presented in that way when there was no break point to refer to, according to CLSI.

**Table 4:** MIC values of bacteria isolated from the uteruses of 30 donkeys at ECDC between 2018 and 2021.

Bacteria	*E. coli*		*Acinetobacter* spp.		*P. aeruginosa*		*Klebsiella* spp.		*SEZ*	
MIC Values (μg/mL)	MIC_50_	MIC_90_	MIC_50_	MIC_90_	MIC_50_	MIC_90_	MIC_50_	MIC_90_	MIC_50_	MIC_90_
Cefoxitin	-	-	-	-	>128	>128	-	-	<0.25	<0.25
Ceftiofur	-	-	-	-	32	32	-	-	-	-
Kanamycin	-	-	-	-	>128	>128	-	-	32	32
Enrofloxacin	<0.25	1	<0.25	1	8	8	<0.25	<0.25	1	1
Rifampicin	4	8	<0.25	0.5	>128	>128	16	64	-	-

## Data Availability

Data available in a publicly accessible repository. The data presented in this study are openly available in FigShare at https://doi.org/10.6084/m9.figshare.18979376, accessed on 1 January 2022.
